# Conditioned Placebo- and Nocebo-Like Effects in Adolescents: The Role of Conscious Awareness, Sensory Discrimination, and Executive Function

**DOI:** 10.3389/fpsyt.2020.586455

**Published:** 2020-11-19

**Authors:** Regula Neuenschwander, Ella Weik, Christine M. Tipper, Karin Jensen, Tim F. Oberlander

**Affiliations:** ^1^Department of Pediatrics, BC Children's Hospital Research Institute, University of British Columbia, Vancouver, BC, Canada; ^2^Institute of Psychology, University of Bern, Bern, Switzerland; ^3^Department of Psychiatry, BC Mental Health and Substance Use Services Research Institute, University of British Columbia, Vancouver, BC, Canada; ^4^Department of Clinical Neuroscience, Karolinska Institute, Stockholm, Sweden

**Keywords:** conditioning, sensory discrimination, nocebo effect, executive function, youth, thermal perception

## Abstract

**Background:** Conditioning is a key mechanism of placebo and nocebo effects in adults. Little is known about the underlying mechanisms of placebo and nocebo effects in youth and how they might be influenced by conscious awareness and cognitive abilities. In this study, the role of conditioning on thermal perception in youth was investigated.

**Methods:** Differences in thermal ratings were assessed in response to consciously and non-consciously perceived cues that were conditioned to either low or high heat. Furthermore, we tested whether executive function mediates the effect of conditioning on thermal perception. Thirty-five high-school students (14–17 years) completed an executive function task and underwent a sensory perception paradigm. In a conditioning phase, two distinct neutral faces (conditioned cues) were coupled to either a low or a high temperature stimulus delivered to participants' forearms. In a testing phase, the conditioned cues, and novel faces (non-conditioned control cues), were paired with identical moderate thermal stimuli. In this testing phase, for half of the participants cues were presented consciously (supraliminally) and for the other half non-consciously (subliminally).

**Results:** We found a significant main effect of cue type on thermal ratings (*p* = 0.003) in spite of identical heat being administered following all cues. *Post-hoc* analyses indicated that the nocebo-like effect (conditioned high cue compared to control) was significant (*p* = 0.027); the placebo-like effect (conditioned low cue compared to control) was non-significant. No difference between cues presented supra- vs. subliminally and no significant interaction effects were found. The association between sensory discrimination and the magnitude of the nocebo-like effect was mediated by executive function.

**Conclusions:** To our best knowledge, this is the first study establishing a relationship between thermal perception, nocebo effects, and executive function in youth. Our results may have important implications for understanding cognitive/ learning processes involved in nocebo effects.

## Introduction

Placebos can induce pain relief similar to analgesic drugs in both adults and adolescents (1, 2). One key mechanism of placebo effects is conditioning (3). In conditioning paradigms, the sensory experience of pain and pain relief are systematically and repeatedly paired with distinct cues. In a subsequent testing phase, pain cues can increase subjective discomfort of physical stimulation (nocebo-like effect), whereas pain relief-related cues can decrease it (placebo-like effect) (4–7). This may be explained in terms of predictive coding, representing the brain's ability to optimize sensory processing by integrating predictive cues and prior experience (8).

In adults, placebo and nocebo effects have been associated with activity in prefrontal brain regions (9). Likewise, disrupted placebo effects were observed when prefrontal areas were inhibited (10, 11) or impaired (12). Activity in frontal brain regions associated with placebo-/nocebo-like effects might therefore reflect involvement of higher-order cognitive abilities [i.e., executive function including working memory, inhibitory control, and shifting/cognitive flexibility (13)]. Executive function has been defined as the use of (higher-order) cognitive processes to direct, engage, and coordinate other (lower-order) cognitive processes, typically involving the deliberate control of thoughts and actions (14).

One key predictor of the strength of conditioned placebo and nocebo effects is sensory discrimination (5), which is the ability to discriminate between different intensities of sensory experiences. Spearman has suggested over a century ago that sensory discrimination is associated with higher cognitive functioning (15). In more recent years, this association has been supported by findings that higher intelligence test-scores correlated with better visual, auditory, and tactile discrimination in school children (16–18). Importantly, a few studies have specifically looked at this association with tasks related to executive function (17, 18). Although, Spearman himself (19) did not assume that sensory discrimination was more basic and therefore a cause of differences in intelligence (he assumed “some deeper fundamental cause” was underlying these two variables), to date significant correlations between sensory discrimination and intelligence have usually been seen as a support of the hypothesis that intelligence is based on various basic processes, such as processing speed and accuracy as well as sensory discrimination [e.g., (17, 20)]. We will build on this body of research, by conceptualizing executive function as a higher-order cognitive process (like cognitive processes measured in intelligence tests) that is predicted by sensory discrimination of heat stimuli (a basic process) and is itself predictive of conditioned placebo-/nocebo-like effects (a lower-order cognitive process).

In contrast, however, conditioning can be induced without conscious awareness (5, 21, 22), speaking against executive function as a prerequisite for conditioned pain responses. Furthermore, a recent meta-analysis found that the magnitude of the placebo response correlated with IQ in patients with genetically determined intellectual disabilities (23), indicating that in spite of severely impaired cognitive functions, patients with intellectual disabilities (e.g., Down's syndrome) showed placebo responses. Thus, the role of higher-order cognitive processes (i.e., executive function) in conditioned placebo-/nocebo-like effects remains inconclusive. Here, we aim to explain the relation between sensory discrimination and the nocebo-/placebo-like effect by executive function. This leads to the question of whether executive function mediates the association between sensory discrimination and conditioned placebo- and nocebo-like effects.

While placebo mechanisms have been studied intensively in adults, studies in adolescents are still rare and show conflicting results (24–27). Wrobel et al. (27) used a placebo heat paradigm involving both a combination of conditioning and expectation (i.e., analgesic cream that was de facto inert) to induce placebo effects in children/youth and adults. They found a significant placebo effect in both groups. While the magnitude of the placebo effect did not differ between children/youth (10–15 years) and adults, it was predicted by prior experience (cf., sensory discrimination) in children/youth only. Gniß and colleagues (26) aimed to disentangle conditioning from expectations by investigating them separately with a heat paradigm in children (younger group: 6–9 years; older group: 10–13 years), youth (14–17 years), and adults (>18 years). Of particular interest, for the conditioning paradigm they applied a placebo cream and lower heat was conditioned to one randomly chosen arm (i.e., cream). In contrast to Wrobel et al. (27), they only found a significant placebo effect in children in the conditioning paradigm, but no effect in youth or adults with small effect sizes for youth and adults, and moderate ones for children. Sensory discrimination predicted the magnitude of the placebo effect in all age groups. Similar to Wrobel et al. (27), the size of the correlation, however, decreased with age. While these two studies show conflicting results, both imply that developmental aspects have an influence on induced placebo effects.

Here, we aim to add to this literature by applying a well-established sensory perception paradigm previously shown to induce placebo-like analgesia and nocebo-like hyperalgesia in adults (5, 21) to an adolescent population (14-17 years). We induced both placebo- and nocebo-like effects purely by conditioning rather than conditioning to a placebo cream, which is a combination of expectation and conditioning. Additionally, to date, no study has investigated how conditioning of placebo- and nocebo-like effects might be influenced by adolescents' executive function. Adolescence is a sensitive period for brain development and the maturation of executive function (28, 29), which may have crucial implications for our understanding of conditioned placebo- and nocebo-like effects in youth.

In sum, in the present study we investigate: (1) modulation of thermal perception (subliminally or supraliminally) by exposure to visual cues conditioned to low or high thermal stimulation in youth and (2) the role of executive function. Based on previous research, we expect to find similar results in modulation of thermal perception as were found in adults. Specifically, we hypothesize that moderate temperatures paired with conditioned high heat cues will be perceived by adolescents as more uncomfortable (nocebo-like effect) and conditioned low heat cues will be experienced as less uncomfortable (placebo-like effect), compared to neutral cues. Further, we predict that adolescents' placebo-/nocebo-like effects will be weaker with subliminal compared to supraliminal cue presentation. Finally, we expect that effects of sensory discrimination on placebo-/nocebo-like effects will be mediated by executive function.

Since youth represent a vulnerable population, uncomfortable (but not painful) thermal sensations served as a model for pain and were used to tap into placebo-/nocebo-related learning mechanisms and pain-related processes in this study.

## Methods

### Participants

*N* = 35, 14- to 17-year-old adolescents (74.3% males, *M* = 16.13 years, *SD* = 0.84) were recruited from a local high school in Vancouver, BC, Canada. Individuals with the presence of any illness or medication use that was judged to interfere with the study, such as psychiatric disorders according to the DSM-V manual, medication that can influence cognition or emotional processing, i.e., sleep medication, antidepressants, anti-convulsant or opioids were excluded from the study. *N* = 17 participants were assigned to the conscious exposure group (supraliminal: cues presented with awareness), *n* = 16 participants were assigned to the non-conscious exposure group (subliminal: cues presented without awareness), and *n* = 2 participants had to be excluded from statistical analyses due to non-compliance during experiment.

### Procedure

The study was approved by the University of British Columbia, Children's & Women's Health Centre of BC Research Ethics Board. After parents and participants had given consent, participants completed a computer-based executive function task (Reversed Flanker). Afterwards they completed the sensory perception paradigm to assess the influence of conditioning on thermal sensations (placebo- and nocebo-like effects). Participants received a 20 dollar gift card for their participation and were debriefed in the context of a neuroscience teaching curriculum after all participants had completed the experiment.

### Material

The Reversed Flanker task was used to assess two of three key components that comprise executive function: inhibitory control and cognitive flexibility (30–32). This is a widely used computerized executive function measure that has been validated with 4-year-olds through adults and depends on lateral prefrontal cortex and interrelated structures. During the task, participants were asked to focus on a fish in the middle of the screen (target) and ignore the distractor fish on either side. During the first block, participants were asked to feed the middle fish by pressing where it was facing (e.g., press left button if middle fish is facing to the left). In the second block, participants were asked to focus on the outside fish and press where they were facing, while ignoring the middle fish. During the third block, participants had to switch between these two versions of the task. The conditions of interest were whether the target fish matched the direction of the distractor fish (congruent), or did not match (incongruent). Blocks 1 and 2 consisted of 17 trials each and block 3 consisted of 65 trials, with an inter-trial interval of 500ms. Stimuli were presented for 1,500 ms. Responses >2,000 ms were considered incorrect (inattentive) and those <250 ms impulsive (too fast to have been in response to the stimulus) and excluded from the analysis. Outlier trials were removed by using a lower and upper threshold of two standard deviations from the mean response time (RT) per trial type per block and per subject. The Flanker effect was defined as RT incongruent—RT congruent in the second block (i.e., the higher the Flanker effect, the poorer the executive function). Importantly, block 2 assesses inhibitory control (ability to inhibit visual distraction) as well as cognitive flexibility (ability to inhibit an old strategy and switch to a new one).

Thermal sensations were induced on the left volar forearm with the Thermal Sensory Analyzer, using a 3 cm × 3 cm probe (Medoc Advanced Medical Systems, Rimat Yishai, Israel; Biomedical Engineering Device). The sensory perception design was adapted from Jensen and colleagues (5). Stimuli were presented for 4 s with a ramp up and ramp down period of 8°/s. Participants first underwent a calibration phase in which an individual high and low thermal stimulus was calibrated. Calibration started at 36°C. Each temperature was presented three times and then raised up 1°C higher. The temperature, which was rated as a discomfort level of 60 on a visual numeric scale between 0 (“no discomfort”) and 100 (“worst imaginable discomfort”) was used as high thermal stimulus. The low thermal stimulus was calculated by subtracting 3°C from the high stimulus. The moderate thermal stimulus was halfway between the low and high stimulus. As youth represent a vulnerable population it was deemed more suitable to use the wording “thermal discomfort” rather than “pain” during the experiment. Moreover, the participants were explicitly told that we do not want the stimuli to cause any pain and asked them to verbalize if the stimulus was getting painful. Twelve distinct black-and-white pictures of middle-aged Caucasian male faces (neutral facial expression) from the Karolinska Directed Emotional Faces (KDEF) package were used as visual cues (33). During the conditioning phase, the low and high heat stimulus was repeatedly paired with two different faces, i.e., the conditioned cues. During the testing phase, moderate thermal stimuli where paired with the conditioned visual cues as well as four different non-conditioned control cues. In the supraliminal cue group, conditioned cues were presented long enough (100 ms) for participants to be consciously aware of the cue and recognize the face. In the subliminal cue group, conditioned cues in the testing phase were presented very quickly and subsequently masked, such that they could not be consciously recognized (note that cues were presented supraliminally in the conditioning phase). The face was presented for 12 ms and then masked by a scrambled version of the picture.

The conditioning phase consisted of 40 trials (20 cue-stimulus pairing for each condition) and was presented in two blocks. The testing phase consisted of 60 trials (20 for high temperature, 20 for low temperature and 20 for neutral cues), spilt into three blocks with 20 trials each. Two high temperature cues and two low temperature cues in each of the three blocks were paired with their original conditioned temperatures, to prevent extinction. These “booster” trials as well as the first trial of each block were not included in the statistical analyses. Between blocks, participants were able to take a short break. For each run the inter-trial interval was 3–5 s. Participants' discomfort evoked by the heat was assessed with verbal ratings on a visual numeric scale between 0 and 100, ranging from “no discomfort” to “worst imaginable discomfort.” A post-test was performed where we presented participants 24 faces (six “old” faces and six “new” faces, with two exposures each = 24 exposures in total) and asked them “Have you seen this face before during the experiment?”

The main outcome variable was the subjective rating of discomfort in response to thermal stimuli. Sensory discrimination was determined as the difference between the mean discomfort rating of high and low heat stimuli during the conditioning phase. In the testing phase, differences in mean discomfort in response to moderate temperatures paired with either “low heat” cues, “high heat” cues and control cues were calculated. The magnitude of conditioned analgesia (placebo-like effect) was estimated by the difference in mean discomfort for control and low heat cues. The magnitude of conditioned hyperalgesia (nocebo-like effect) was estimated by the difference between mean discomfort for high heat and control cues. The magnitude of the conditioning effect was calculated by the difference in mean discomfort of low and high heat cues.

### Statistical Approach

The influence of conditioning on thermal discomfort ratings was analyzed using a 3 × 2 mixed-model ANOVA with “cue type” (high conditioned, low conditioned, or neutral) as within factor and awareness as between subject factor. *Post-hoc* comparisons were conducted were appropriate (main effect “cue type“) with Fisher's least significant difference (LSD) test as this method is more powerful than using Bonferroni when *k* = 3 as Meier has shown that Fisher's LSD test, “preserves the experimentwise type I error rate at the nominal level of significance, if (and only if) the number of treatment groups is three” [page 253 in (34)]. Furthermore, a mediation analysis was conducted in order to test if executive function can explain the effect of sensory discrimination on the magnitude of the placebo- and nocebo-like effect. For these analyses, we used the “indirect” macro designed for SPSS (35). This procedure is well-suited for small sample sizes and accounts for the possibility of non-normality and/or asymmetry for the indirect effect. Importantly and contrary to the more traditional causal steps logic which requires that all three paths (a, b, and c) are statistically significant (36), individual paths are not required to be significant in order to determine whether M (in our model, executive function) mediates the effect of X (in our model, sensory discrimination) on Y (in our model, nocebo-like effect) (37). Instead, all that is required is that ab (i.e., indirect effect) is statistically different from zero. For all indirect paths, parameter estimates and 95% confidence intervals (CI) were derived and mediation is supported when the CIs do not contain zero.

## Results

### Preliminary Analyses

#### Manipulation Check

Post-test analyses revealed significant group differences, indicating that the supraliminal group had more correct answers (*M* = 15.47, *SD* = 3.4) than the subliminal group (*M* = 12.31, *SD* = 3.4), *F*(1,32) = 7.0, *p* = 0.013. Furthermore, participants in the subliminal group performed at chance level, which suggests that the subliminal cues during the testing phase were not consciously perceived.

#### Group Comparisons

As we assessed awareness (subliminal vs. supraliminal presentation of cues during the testing phase) as a between subject factor, group comparisons on crucial variables are presented here. Gender was balanced across groups (12 males and 5 females in supraliminal; 12 males and 4 females in subliminal). There was a small but significant difference in participants' age across groups *t*(31) = 2.10, *p* = 0.044 (supraliminal: *M* = 15.85 years, subliminal: *M* = 16.39 years). Importantly, there were no significant group differences on heat ratings during the calibration and conditioning phase: calibration phase, *t*(31) = 0.61 *p* = 0.547 (supraliminal: *M* = 46.24°C, subliminal: *M* = 45.94°C) as well as discomfort ratings of high heat in the conditioning phase: *t*(31) = 0.34, *p* = 0.738 (supraliminal: *M* = 43.51°C, subliminal: *M* = 45.42°C). Finally, there were no group differences in the Flanker effect: *t*(31) = 0.64, *p* = 0.525 (supraliminal: *M* = 12.44, subliminal: *M* = −5.73).

### Thermal Sensations

Average temperatures obtained to elicit high heat ratings were *M* = 46.09°C (*SD* = 1.40°C). Note that moderate and low heat cues were calculated by subtracting 1.5 and 3°C, respectively, from the high heat stimulus, resulting in *M* = 44.59°C for moderate heat cues and *M* = 43.09°C for low heat cues.

**Figure 1:**
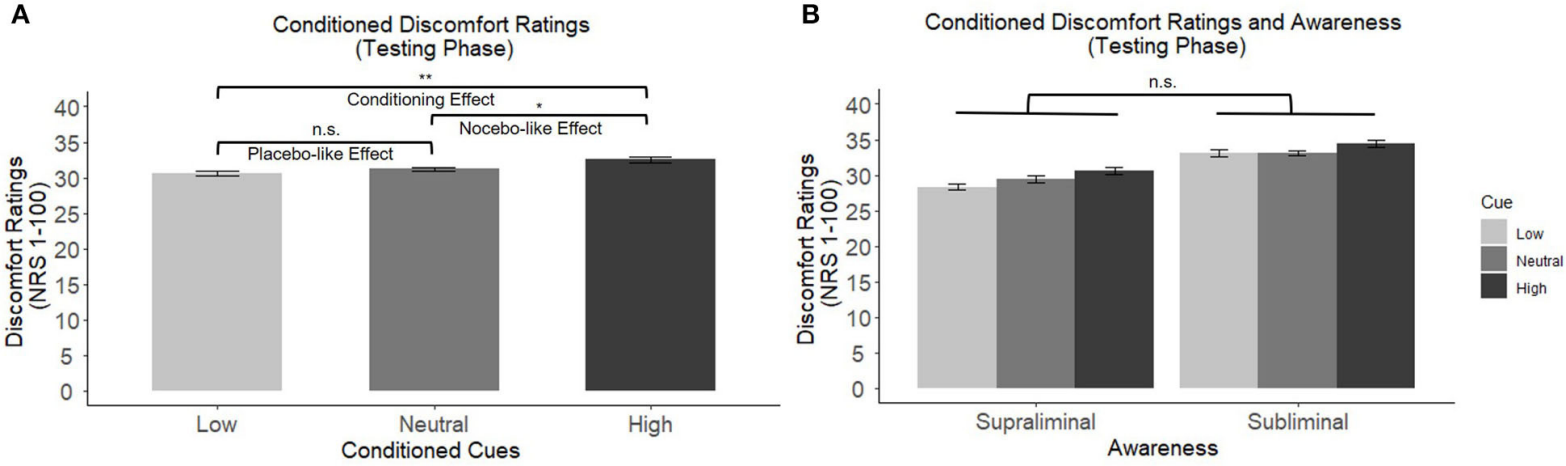
**(A)** Main effects of conditioned cues on discomfort ratings of moderate temperatures. *Post-hoc* comparisons revealed a significant conditioning effect and a significant nocebo-like effect. **(B)** No significant difference between cues presented supra- vs. subliminally (between subject factor “awareness”). *p < 0.05, **p < 0.01, ***p < 0.001. n.s., not significant.

During the conditioning phase participants rated the low heat stimulus as significantly less uncomfortable (*M* = 24.69, *SD* = 15.05) compared to the high heat stimulus (*M* = 44.44, *SD* = 16.25), *F*(1,32) = 106.4, *p* < 0.001, meaning participants were able to discriminate between the different temperatures. The difference between the high and low temperature rating will hereby defined as sensory discrimination. During the testing phase, when only moderate heat stimuli were delivered, a 3 × 2 mixed-model ANOVA showed a significant main effect of conditioned cues on thermal ratings (*F*(2,62) = 6.51, *p* = 0.003, see Figure 1A), with no difference between cues presented supra- and subliminal (*F*(1,31) = 0.38, *p* = 0.540, see Figure 1B), and no significant interaction effects. *Post-hoc* comparisons (Fisher's LSD test) showed a significant conditioning effect, as there was a significant difference between the heat ratings of moderate thermal stimuli that followed low and high conditioned cues (*p* = 0.003). Furthermore, there was a significant nocebo-like effect (high vs. neutral cue, *p* = 0.025). The placebo-like effect (low vs. neutral cues), however, did not reach statistical significance (*p* = 0.192).

**Figure 2:**
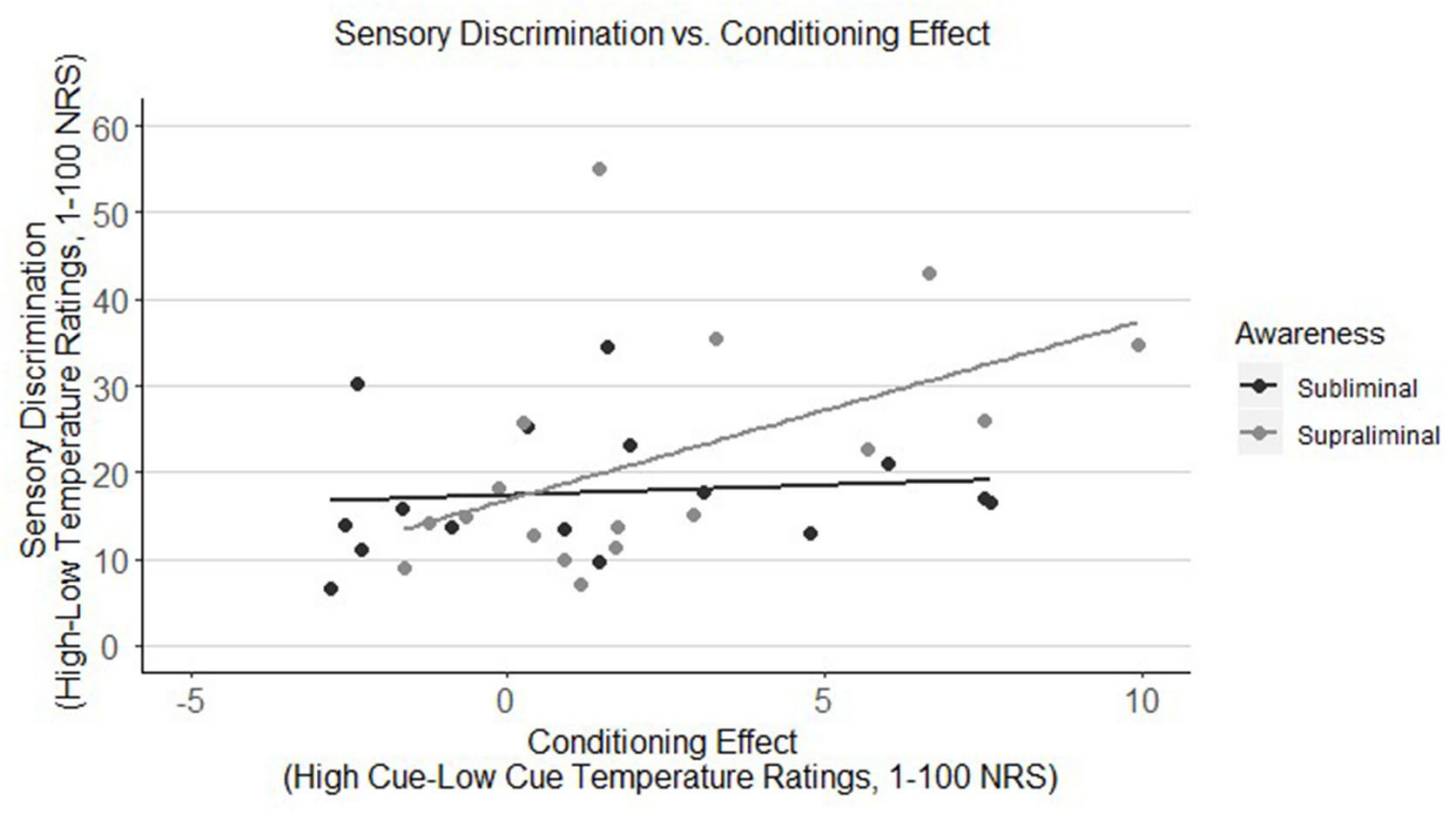
Correlation of sensory discrimination of thermal stimuli with the conditioning effect in youth by awareness group (subliminal vs. supraliminal). Sensory discrimination is only correlated with the conditioning effect when stimuli were presented supraliminally (*r* = 0.50, *p* = 0.039), meaning that adolescents in this group who were better able to differentiate between high and low heat stimuli, showed a higher conditioning effect. Adolescents in the subliminal group, however, did not show this relationship (*r* = 0.11, *p* = 0.691). NRS, numeric response scale.

As represented in Figure 2, there was a significant positive correlation between sensory discrimination and the conditioning effect, but only when stimuli were presented supraliminally (*r* = 0.50, *p* = 0.039), meaning that the better the discrimination between heat stimuli during the conditioning phase, the greater the effect of the conditioned cues during the testing phase. Adolescents in the subliminal group, however, did not show this relationship (*r* = 0.11, *p* = 0.691).

### Executive Function

**Figure 3:**
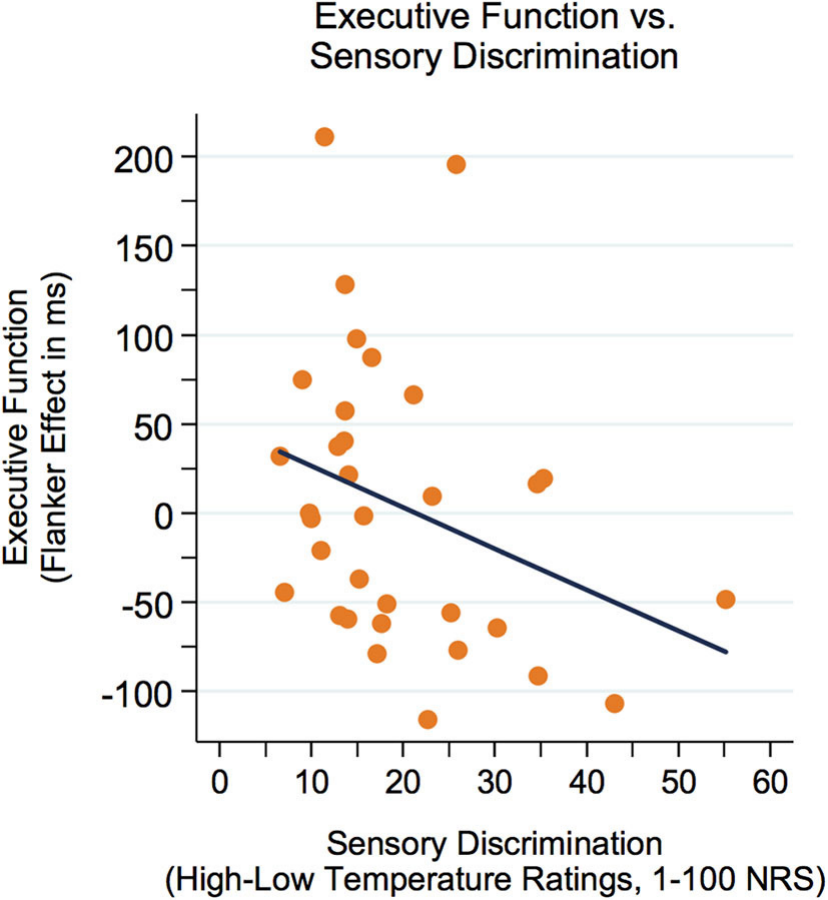
The Flanker effect was modestly yet not significantly correlated with sensory discrimination (*r* = −0.32, *p* = 0.073). Better executive function = lower Flanker effect. NRS, numeric response scale.

**Figure 4:**
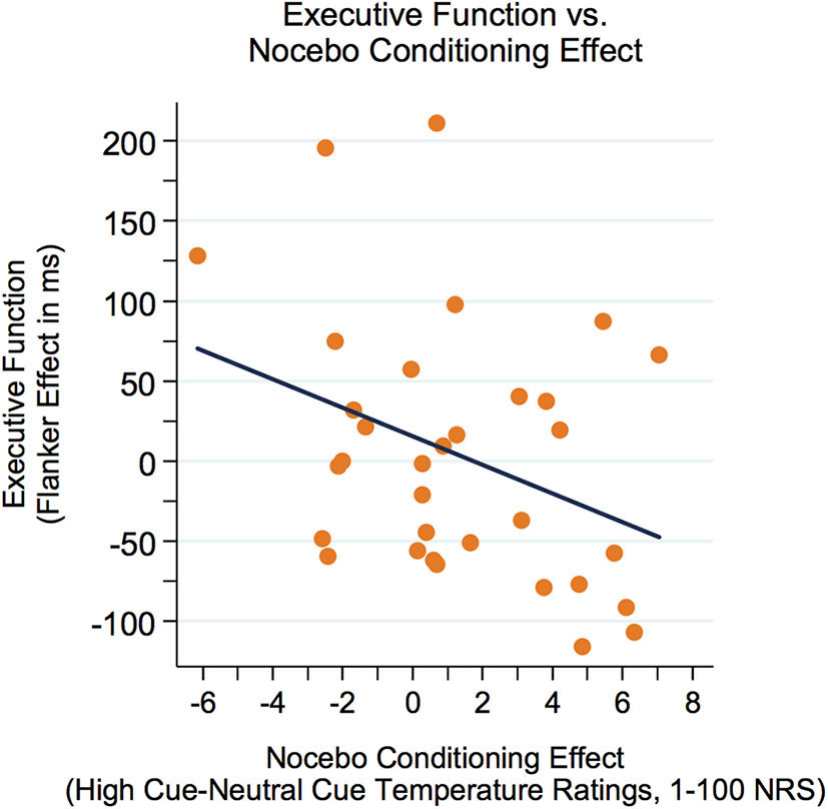
The Flanker effect was negatively related to the nocebo-like effect (*r* = −0.36, *p* = 0.042), suggesting that adolescents with better executive function (lower Flanker effect) showed a higher nocebo-like effect. Better executive function = lower Flanker effect. NRS, numeric response scale.

First, correlations between executive function (Flanker effect) and variables of sensory perception were examined. As represented in Figure 3, there was a modest yet not significant negative correlation between the Flanker effect (lower Flanker effect = better executive function) and sensory discrimination (*r* = −0.32, *p* = 0.073). Furthermore, there was a significant negative correlation between the Flanker effect and the nocebo-like effect (*r* = −0.36, *p* = 0.042, see Figure 4), suggesting that adolescents with better executive function (lower Flanker effect) showed a higher nocebo-like effect.

A mediation model was run to investigate whether executive function (Flanker effect) could explain the association between sensory discrimination and the nocebo-like effect. Importantly, for sensory discrimination as well as for the nocebo-like effect, we only included block 1 of the conditioning and testing phase, respectively, in order to have a more unbiased/clean perception of the heat stimuli, assuming that in later blocks residual heat affects participants' ratings.

**Figure 5:**
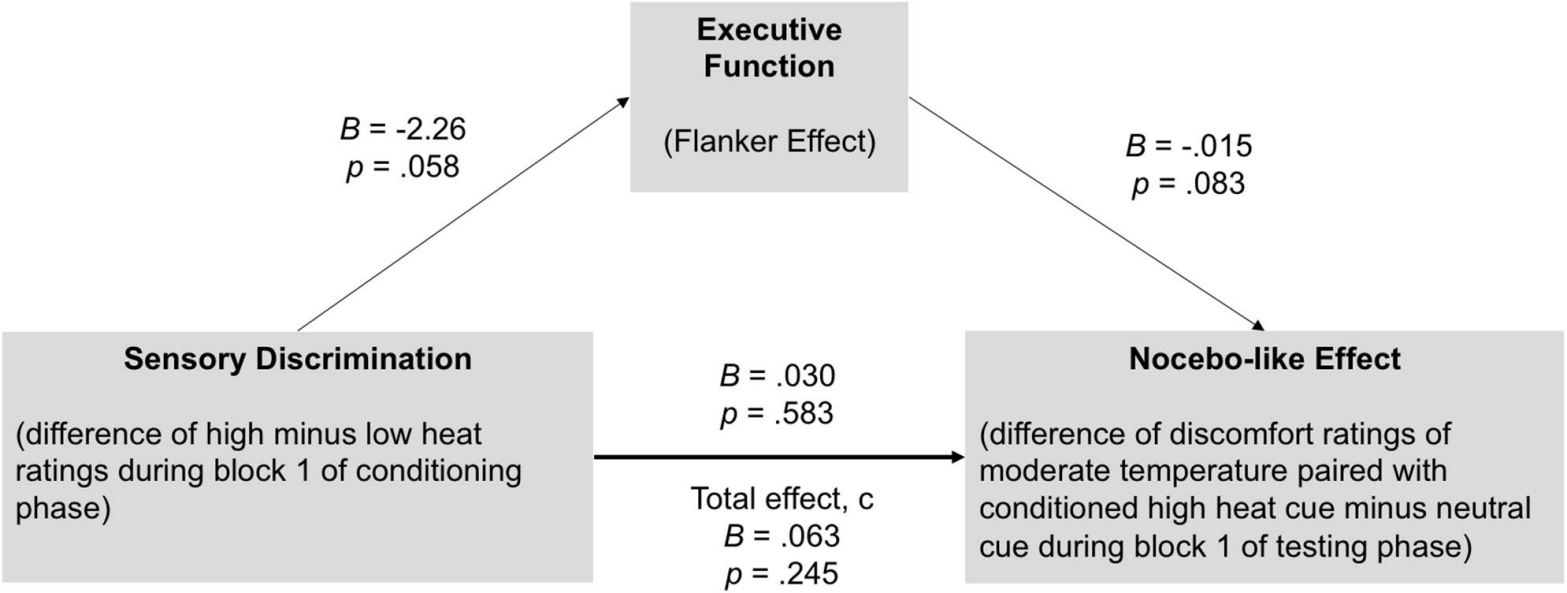
Executive function (Flanker effect) mediates the association between sensory discrimination and the nocebo-like effect in youth. There was a significant indirect effect of sensory discrimination ➔Flanker effect ➔nocebo-like effect, *B* = 0.033 (0.028) 95% CI [0.006–0.1173], suggesting that executive function partially explain the association between sensory discrimination and the magnitude of the nocebo-like effect. Unstandardized estimates.

As represented in Figure 5, there was a significant indirect effect of sensory discrimination ➔Flanker effect ➔nocebo-like effect, *B* = 0.033 (0.028) 95% CI [0.006–0.1173]. The total effect c was *B* = 0.063, *p* = 0.245, and dropped to *B* = 0.030, *p* = 0.583 (direct effect c') when the mediator (Flanker effect) was included in the model. These results indicate that executive function mediated the association between sensory discrimination and the magnitude of the nocebo-like effect.

## Discussion

In line with conditioned placebo- and nocebo-like effects in adults, the results from the present study demonstrate that conditioning can change the perception of thermal discomfort in youth. Conditioning is one of the key mechanisms of placebo and nocebo effects in adults. Placebo and nocebo responses are apparent in children and youth in clinical trials, it however remains unclear whether the underlying mechanisms of these effects in youth are the same as those in adults. In this study we replicated a conditioning paradigm previously shown to induce placebo-like analgesia and nocebo-like hyperalgesia in adults (5, 21), in an adolescent population. Adolescents rated moderate thermal sensations as more uncomfortable when paired with a conditioned high cue compared to a conditioned low cue. The difference in the discomfort ratings, however, was small. Contrary to the adult studies by Jensen and colleagues (5, 21), only comparison of the high discomfort vs. neutral control cue ratings (nocebo-like effect) was significant, and the low discomfort vs. control cue ratings (placebo-like effect) did not reach significance. While there was an overall conditioning effect evidenced by a significant difference in thermal ratings of moderate thermal stimuli following low and high conditioned cues, our results suggest that conditioning of thermal perception is not as effective in adolescents as in adults, at least not in the present experimental setup. Of particular interest, though, we found that the effect of sensory discrimination ability on nocebo-like effects was mediated by adolescents' executive function. This suggests that executive function, and in particular inhibitory control and cognitive flexibility, may play a major role in classical conditioning of thermal perception in youth. In line with the adult data from Jensen and colleagues (38), there was no difference in conditioned effects related to level of conscious awareness of the conditioned cues. However, sensory discrimination was only related to the conditioning effect when stimuli were presented supraliminally as opposed to subliminally, indicating that the level of conscious awareness may play a role in conditioning of placebo-/nocebo-like effects in youth, even if it did not affect these effects directly.

While we were able to induce significant nocebo-like effects, the effects were much smaller compared to the adult studies applying the same paradigm, which showed large effect sizes [cf., η^2^ as an indicator of the proportion of variance accounted for by placebo-/nocebo-responses triggered by conditioning–Jensen et al. (5): η^2^ = 0.57; Jensen et al. (21): η^2^ = 0.41, our study, η^2^ = 0.17]. It is possible that more trials are needed for youth to obtain similarly strong placebo-/nocebo-like effects as in adults. Interestingly, in adults, fewer trials are necessary to induce conditioned nocebo-like effects compared to placebo-like effects (39), which may explain why the nocebo-like but not the placebo-like effect reached level of statistical significance.

Various reasons, however, may account for non-significant placebo-like effects. This is in line with a recent study by Gniß and colleagues (26), who did not find significant placebo effects (induced by conditioning and expectation) in the adolescent subpopulation with regards to subjective pain reports. In contrast, they found almost twice as high effect sizes for younger and older children as compared to adolescents. It is also possible that our adolescent population perceived thermal sensations more precisely than adults and were therefore less influenced by the conditioned cues. It is also possible that more conditioning trials are needed for the adolescent population to establish a cue-stimulus association than for adults. Another explanation may be found within the framework of Bayesian estimates or predictive coding aiming to understand placebo- and nocebo-like effects (8, 40, 41). Contextual variables, such as conditioned cues, can facilitate efficient processing of incoming noisy sensory experiences. In order to establish a predictive model of the sensory environment, sustained attention toward cues and sensory experiences, as well as the ability to distinguish between threat and safety cues, are required during conditioning. Interestingly, both of these mechanisms are still maturing during adolescence. Thillay et al. (42) showed that the performance in a visual detection task increased with age and that this effect is most likely due to developmental immaturity of frontal brain regions associated with sustained attention during adolescence (42). Thus, it may be possible that the adolescent participants in our study had difficulties to sustain attention toward the presented cues during the learning sequence and therefore did not establish a predictive model of the sensory experiences.

In line with our findings, previous literature also shows that adolescents compared to adults showed smaller differences between reported fear ratings in response to safety and threat cues during a threat-learning paradigm (43). Furthermore, in studies of threat learning, adolescents have been shown to recruit subcortical structures during the conditioning phase, and showed a negative correlation between prefrontal activity and the strength of fear learning, whereas adults recruited prefrontal areas and the activity in these areas was associated with higher fear during the learning phase (43). Of particular interest, in adults, interfering with prefrontal brain activity with transcranial direct current stimulation after the condition phase abolished placebo and nocebo effects in the testing phase (10). As prefrontal areas are crucially changing during adolescence, the ability to induce meaningful conditioned placebo- and nocebo-like effects might be compromised in youth.

Nevertheless, we also found that better executive function was associated with higher nocebo-like effects. In particular, executive function partially mediated the association between sensory discrimination and the magnitude of the nocebo-like effect. This is supported by previous studies reporting positive associations between better executive function and sensory modalities such as smell [odor discrimination (44)], vision [visual discrimination (17, 18)], hearing [pitch discrimination (18)], and touch [haptic weight discrimination (17)] on the one hand, and previous research showing that IQ predicted the strength of the placebo effect in patients with intellectual disabilities on the other hand (23). To the best of our knowledge, this is the first study, to establish a relationship between thermal perception and executive function in youth, which may have important implications for our understanding of cognitive/learning processes involved in pain and nocebo effects. Further investigation, however, is needed to corroborate our findings and possibly also test alternative accounts of the relationship between these three variables.

### Limitations and Future Studies

Given that youth is considered a vulnerable population in terms of research ethics, we used uncomfortable, but not painful, thermal sensations as a model for pain. While we might not be able to make direct conclusions about pain *per se*, we nevertheless believe that our findings provide insights into placebo-/nocebo-related learning mechanisms in youth [especially since the calibrated temperature range in our youth sample [43–49°C] was similar to the range in adults [44–50°C] in Jensen et al. (5)].

Even though conditioning changed sensory perception in adolescents, the effect sizes were small. The observed differences are thus not in the range of so-called clinical validity. Of particular interest may also be the fact that almost a third of our participants can be considered placebo/nocebo non-responders, which is in line with Gniß et al. (26) study who report almost 20% of “non-learning” participants, mostly children, in the conditioning paradigm. Furthermore, we attempt to make assumptions about differences between adolescents and adults. While using a study design that was previously used in adults, we did not include an adult population in the current study, so we cannot rule out the possibility that differences in our data might be due to differences in the study design or execution rather than differences between adults and youth. While this assumption was based on previous literature from other research groups, brain imaging studies examining maturation effects in conditioning-related prefrontal and pain-mediation brain pathways are required to support these theoretical considerations. Additionally, the use of verbal ratings for subjective discomfort might have biased participants' answers. We therefore cannot rule out demand effects, i.e., participants may have inferred the purpose of our study and responded so as to help confirm our hypotheses. Adding more objective measurements such as skin conductance or facial expression might be beneficial in obtaining more reliable estimates of placebo-/nocebo-like effects. Future studies should also address whether placebo-/nocebo-like effects can be enhanced by increasing adolescents' attention toward the cues, or by using more distinct cues so as to facilitate conditioning to each cue. Finally, the mediating effect of executive function on the association between sensory discrimination and the nocebo-like effect observed here should be interpreted cautiously. Sensory discrimination, as measured in this study, is not independent of the placebo/nocebo learning (i.e., conditioning learning impacts the ratings later in the conditioning phase). However, to mitigate this concern, we only included the first block of the conditioning phase in our mediation analyses. This decision was based on the assumption that block 1 of the conditioning phase was less affected by conditioning learning than block 2. Future studies, however, may calibrate both low and high temperatures separately, in order to use that range as a measure of discriminability and thus being able to assess sensory discrimination independent from the placebo/nocebo testing phase. Future studies should also replicate the mediation found in this study in the context of observing more robust nocebo effects in youth.

In this study, a conditioning paradigm was used to investigate mechanisms underlying placebo- and nocebo-like effects on thermal perception in youth. Our study demonstrated that conditioned cues can influence thermal discomfort and specifically induce nocebo-like effects. The effects, however, were small. Executive function mediated the relationship between sensory discrimination and nocebo-like effects. In line with findings in adults, there were conditioned effects both in relation to supraliminal and subliminal presented cues, furthering the notion that implicit cognitive processes, as well as executive function, are implicated in conditioned effects of thermal perception. To the best of our knowledge, this study is the first study to empirically investigate the role of conditioning in placebo- and nocebo-like effects in youth and how these effects might be mediated by executive function. Our results may have important implications for understanding cognitive/learning processes involved in nocebo effects.

## Data Availability Statement

The raw data supporting the conclusions of this article will be made available by the authors, without undue reservation.

## Ethics Statement

The studies involving human participants were reviewed and approved by University of British Columbia, Children's and Women's Health Centre of BC Research Ethics Board, and the Review Board of the Vancouver School Board. Written informed consent to participate in this study was provided by the participants' legal guardian/next of kin.

## Author Contributions

TO, KJ, CT, and RN contributed to the conception and design of the study. KJ contributed method and design features of the study. RN performed the study. RN and EW collected the data. RN organized the database and performed statistical analyses. EW and RN wrote sections of the manuscript. All authors contributed to manuscript revision, read, and approved the submitted version.

## Conflict of Interest

The authors declare that the research was conducted in the absence of any commercial or financial relationships that could be construed as a potential conflict of interest.
